# Effects of Omega-3 Fatty Acids on Muscle Mass, Muscle Strength and Muscle Performance among the Elderly: A Meta-Analysis

**DOI:** 10.3390/nu12123739

**Published:** 2020-12-04

**Authors:** Ya-Hui Huang, Wan-Chun Chiu, Yuan-Pin Hsu, Yen-Li Lo, Yuan-Hung Wang

**Affiliations:** 1 Graduate Institute of Clinical Medicine, College of Medicine, Taipei Medical University, Taipei 11031, Taiwan; A0634@tpech.gov.tw (Y.-H.H.); koakoahsu@gmail.com (Y.-P.H.); 2 Department of Dietetics and Nutrition, Heping Fuyou Branch, Taipei City Hospital, Taipei 10065, Taiwan; 3 School of Nutrition and Health Sciences, College of Nutrition, Taipei Medical University, Taipei 11031, Taiwan; wanchun@tmu.edu.tw; 4 Research Center of Geriatric Nutrition, College of Nutrition, Taipei Medical University, Taipei 11031, Taiwan; 5 Emergency Department, Wan Fang Hospital, Taipei Medical University, Taipei 11696, Taiwan; 6 Department of Biomedical Engineering, National Yang-Ming University, Taipei 11221, Taiwan; doma1118@gmail.com; 7 Department of Medical Research, Shuang Ho Hospital, Taipei Medical University, New Taipei City 23561, Taiwan

**Keywords:** docosahexaenoic acid (DHA), elderly, eicosapentaenoic acid (EPA), omega-3 fatty acid, n-3 PUFAs, sarcopenia

## Abstract

There is increasing evidence showing the role of fatty acids and their derived lipid intermediates in the regulation of skeletal muscle mass synthesis and function. However, the role of omega-3 fatty acids remains unclear. Therefore, we conducted a meta-analysis to evaluate the potential effects of omega-3 fatty acids on sarcopenia-related performances among the elderly. Eligible literature and reports of randomized controlled trials were comprehensively searched from the PubMed, Cochrane Library, ClinicalTrials.gov, and Cumulative Index to Nursing and Allied Health Literature (CINAHL) databases until July 2018. A total of 10 articles were available for the meta-analysis. There were minor benefits for muscle mass gain (0.33 kg; 95% CI: 0.05, 0.62) and timed up and go performance (−0.30 s; 95% CI: −0.43, −0.17). Subgroup analyses regarding muscle mass and walk speed indicated that omega-3 fatty acid supplements at more than 2 g/day may contribute to muscle mass gain (0.67 kg; 95% CI: 0.16, 1.18) and improve walking speed, especially for those receiving more than 6 months of intervention (1.78 m/sec; 95% CI: 1.38, 2.17). Our findings provide some insight into the effects of omega-3 fatty acids on muscle mass, especially for those taking supplements at more than 2 g/day. We also observed that a long period of omega-3 fatty acids supplementation may improve walking speed.

## 1. Introduction

Age-related musculoskeletal decline presents a significant risk for falls in the elderly [1] and is becoming a major public health concern with fast-growing aging populations [2]. Sarcopenia (a loss of skeletal muscle mass and function) is common with advancing age [3], and along with frailty, is associated with severe adverse outcomes, including falls, fractures, hospitalization, and early death [4,5]. Furthermore, people with sarcopenia need substantially more medical care and incur more health-related costs [6]. As such, sarcopenia prevention is crucial in reducing the burden on social care systems and the associated costs.

Physical exercise and nutritional supplementation are currently recommended as preventive measures against the loss of muscle mass, muscle strength, or physical performance [7]. Healthy older persons are advised to maintain a daily protein intake of 1.2–1.5 g/kg body weight, and emphasis is placed on stimulating skeletal muscle anabolism [8]. However, the source and amount of protein intake also affect muscle synthesis and metabolism. Studies have shown that high protein consumption may increase the development of insulin resistance and diabetes [9,10,11], so a high protein intake may not be the best way to prevent sarcopenia in some cases. Such recommendations should be informed by individual dietary patterns and daily lifestyles.

**Figure 1:**
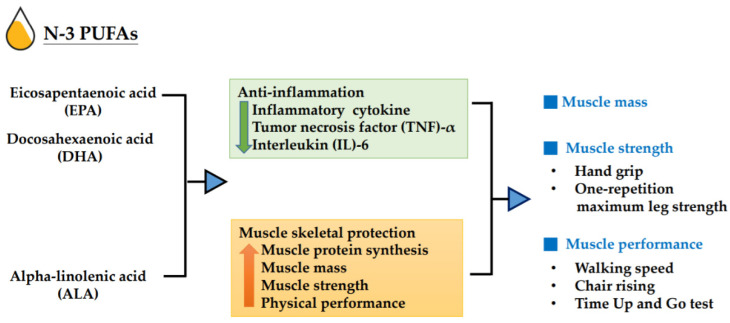
Impact of n-3 polyunsaturated fatty acids (n-3 PUFAs) on muscle mass, muscle strength, and muscle performance.

In addition to protein recommendation, several observational studies and randomized controlled trials (RCTs) report the association of muscle mass and performance with specific nutrients, such as fish-derived n-3 polyunsaturated fatty acids (n-3 PUFAs) [12,13,14,15]. Given that sarcopenia is associated with increased inflammatory responses and impaired glucose homeostasis, recent research suggests that n-3 PUFAs have anti-inflammatory properties, which may be exploited for the prevention or treatment of sarcopenia [16]. Most studies have focused on three main types of n-3 PUFAs: alpha-linolenic acid (ALA, C18:3 n-3), eicosapentaenoic acid (EPA, C20:5 n-3), and docosahexaenoic acid (DHA, C22:6 n-3). Previous evidence [17] showed that n-3 PUFAs from fish, which are high in EPA and DHA, have beneficial effects on cardiovascular health due to their anti-inflammatory properties. An association between n-3 PUFAs intake and musculoskeletal health has also been reported in RCTs [18,19], which showed that supplementation with n-3 PUFAs enhanced the rate of muscle protein synthesis in the elderly; in a strength-training trial [20], fish-oil supplementation resulted in significantly improved muscle strength and functional capacity, compared with those in non-supplemented controls. Furthermore, there is some observational evidence that supports the benefits of fish-oil-derived n-3 PUFAs, from either supplementation with 1.86 g of EPA and 1.5 g of DHA or fatty fish consumption, for muscle mass, muscle strength, and physical function in older people [21,22]. By contrast, a three-year follow-up trial suggested that low-dose n-3 PUFAs (0.225 g of EPA and 0.8 g of DHA) supplementation had no effect on muscle strength in elderly people [23]. ALA is a plant-derived n-3 fatty acid that mainly exists in flaxseed, soybean, perilla, walnut, and canola oils. In healthy adults, only 5–10% and 2–5% of ALA can be converted into EPA and DHA, respectively [24]. A study [25] showed that ALA decreases the levels of plasma inflammatory cytokines, such as tumor necrosis factor (TNF)-alpha and interleukin (IL)-6, which may further improve muscle mass and strength in the elderly. As a modifiable lifestyle factor, n-3 PUFA supplementation is a potential target for preventing sarcopenia in the elderly [26]. The possible mechanism is shown in Figure 1.

The musculoskeletal health benefits of n-3 PUFAs remain inconclusive; thus, the present systematic review and meta-analysis assesses the probable effects of increasing n-3 PUFAs (through supplementation or dietary ingestion) on key skeletal muscle outcomes in adults aged 60 years or older. The investigated outcomes are muscle mass, muscle strength, and muscle performance.

## 2. Materials and Methods

### 2.1. Data Sources and Searches

This systematic review and meta-analysis was performed in accordance with the Preferred Reporting Items for Systematic Reviews and Meta-Analyses (PRISMA) guidelines [27]. We searched PubMed, ClinicalTrials.gov, Cumulative Index to Nursing and Allied Health Literature (CINAHL), and the Cochrane Central Register of Controlled Trials (CENTRAL) in any language, from the date of inception until 31 July 2018. The search included the keywords ‘elderly’, ‘muscle mass’, ‘muscle performance’, ‘muscle strength’, ‘dynapenia’, ‘frailty’, ‘sarcopenia’, ‘polyunsaturated fatty acids’, ‘fish oil’, and synonyms.

### 2.2. Selection Criteria

We included RCTs that evaluated the effect of increasing n-3 PUFAs (through diet or supplementation), on the skeletal muscle mass, muscle strength, or muscle performance of older subjects. The participants included adults aged 60 years or older. A study was eligible for inclusion if it reported changes from the baseline to the last available follow-up for one or more of the following outcomes—muscle mass, muscle strength (such as hand grip), or physical performance including gait speed or time up and go test. Studies were excluded if they did not contain primary data (conference abstracts, meta-analyses, reviews, letters to the editor, and case reports).

### 2.3. Data Extraction and Quality Assessment

The data extracted include the authors, year of publication, study design, sample size, mean age, gender, population, duration of follow-up, period of intervention, exercise, type of n-3 PUFAs and dosage (g/day), muscle mass, muscle strength, and muscle performance outcomes. The primary outcomes of the trials were mean differences in the absolute changes in any measurements of skeletal muscle mass, muscle strength, or physical performance.

The methodological quality of included studies was evaluated according to the Cochrane Collaboration risk-of-bias tool [28]. The level of bias was considered to be high, low, or unclear based on seven domains, namely, (i) random sequence generation, (ii) allocation concealment, (iii) the blinding of the participants and personnel, (iv) the blinding of the outcome assessment, (v) incomplete outcome data, (vi) selective reporting, and (vii) other sources of bias. The result was depicted with a summarized risk-of-bias graph.

The quality of the evidence and strength of recommendation were assessed according to the Grading of Recommendations Assessment, Development, and Evaluation methodology (GRADE). The quality of evidence was classified as high, moderate, low, or very low based on judgments of risk-of-bias, inconsistency, imprecision, indirectness, and publication bias [29].

### 2.4. Data Synthesis and Statistical Analysis

Meta-analyses were performed using Review Manager Version 5.3 (RevMan). The primary analyses assessed the effects of n-3 PUFAs on the primary outcomes. The effect sizes were estimated as mean differences (MDs) or standardized mean differences (SMDs) when different scales were used with their 95% confidence intervals (CIs) and graphs created with a random-effects model. The MD was the absolute difference between the mean values in the two groups. The SMD was calculated as the difference in the mean outcome between groups divided by the standard deviation of outcome among participants [30]. We performed the analyses using a random-effects model to yield more conservative results. For all the analyses, *p* < 0.05 was considered statistically significant.

The forest plot is a graphic representation of the overall pooled results of the meta-analysis. The horizontal lines are the 95% CIs representing the probability that these estimates would occur in 95% of included studies. In addition, we need to assign weights, which were obtained by calculating the inverse of the variance of the treatment effect for individual studies based on their contributions to the pooled estimates. The MD was generally used for continuous outcomes under the inverse-variance (IV) method. Heterogeneity estimates between the studies were described by using Cochran Q (Chi-square test) and I^2^ statistics, with values of 25–49% considered low, 50–74% considered moderate, and 75–100% considered high heterogeneity. For the chi-square test, the degrees of freedom (df) are equal to the number of studies minus one. The RevMan software presents an estimate (Tau^2^) of the between-study variance in a random-effects meta-analysis. The Z test was used to examine the statistical significance of the overall effect. Furthermore, we performed subgroup analyses to explore the heterogeneity of the effect estimates according to participant characteristics (e.g., sex), intervention components (e.g., dosage of n-3 PUFAs over and below 2 g per day), and the duration of intervention. Sensitivity analysis was performed by using the one-study-out method and by restricting the synthesis of the findings to RCTs with low risks of bias. Publication bias was assessed by the visual inspection of funnel plots and the Egger bias test. The latter was performed using StataMP, version 14 (StataCorp; 2015; Stat Statistical Software: Release14; College Station, TX, USA; StataCorp LP).

## 3. Results

**Figure 2:**
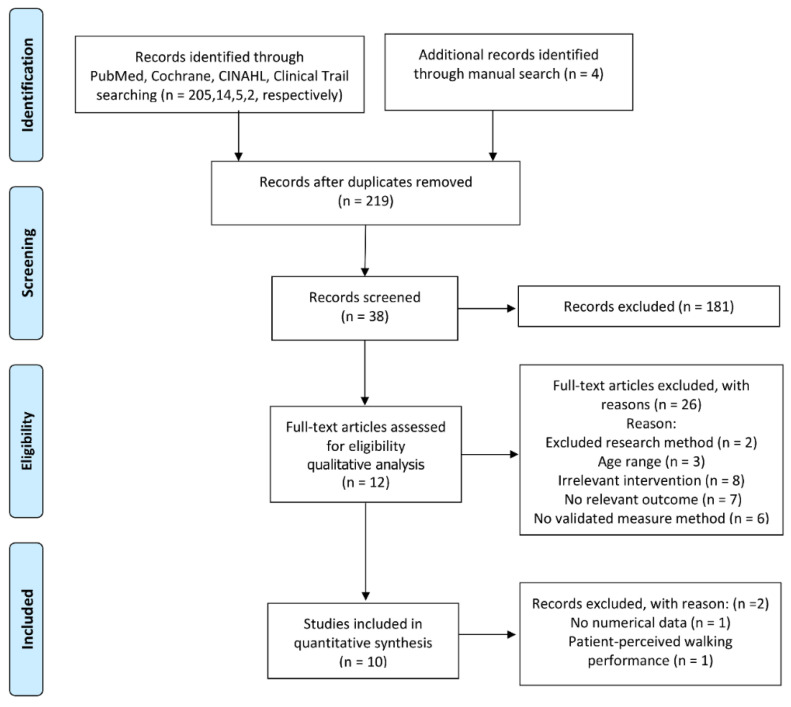
Flow chart of the study selection. Cumulative Index to Nursing and Allied Health Literature (CINAHL).

The screening and selection processes for the included studies are shown in Figure 2. We identified 230 potentially relevant records through multiple database searches (n = 226) and manual searching (n = 4). After excluding duplicate records (n = 11) and irrelevant articles by screening the titles and abstracts (n = 181), thirty-eight studies were evaluated in detail, of which 12 RCTs [20,21,25,31,32,33,34,35,36,37,38,39] (692 participants) met the inclusion criteria. Smith et al. [21] and Grenon et al. [33] were not considered for meta-analysis due to the lack of numerical data for the functional outcomes in the former and the reporting of only patient-perceived walking performance in the latter. Finally, data from 10 RCTs (552 participants) were submitted to the meta-analysis [20,25,31,32,34,35,36,37,38,39].

### 3.1. Characteristics of Eligible Studies

The basic characteristics of the 12 included studies are summarized in Table 1. Among them, five RCTs were conducted in Europe, three in the USA, three in Canada, and one in South America. The number of study participants ranged from 24 to 126, and the durations of the interventions spanned 10 to 24 weeks. Only two RCTs focused on specific diseases (non-small-cell lung cancer and peripheral artery disease); the remaining RCTs included healthy community elderly subjects. Six RCTs included only women, two of which considered only postmenopausal women. The mean ages of the participants across the 12 RCTs ranged from 63 to 75 years old.

Regarding the sources of n-3 PUFAs, nine RCTs provided long-chain n-3 PUFAs (EPA and/or DHA) from fish oil; one RCT provided ALA from flax oil, and two RCTs provided healthy dietary patterns (n-6/n-3 PUFAs < 2). The chemical form of fish oil is generally in the ethyl ester (EE) or triglyceride (TG) type; however, this information may not be formally labeled. Among the nine RCTs that provided n-3 PUFAs from fish oil, only two RCTs mentioned whether the PUFAs were in the EE [21] or TG [31] form.

Strandberg et al. [38] reported a diet plan rich in fish, seafood, whole grains, and vegetables, with limited animal fat and soft drinks, while Edholm et al. [32] reported a diet based on dietary guidelines from Europe and the US; both RCTs monitored the macronutrients and n-6/n-3 PUFAs ratio using food records. Apart from the two RCTs mentioned above [32,38] with diet intervention, only three [20,31,36] of the remaining 10 RCTs requested that the study participants maintain their habitual diet. The doses ranged from 0.16 to 2.6 g/day of EPA and from 0 to 1.8 g/day of DHA. One study provided 14.0 g/day of ALA. Five RCTs reported data combining n-3 PUFA interventions with physical exercise.

### 3.2. Risk of Bias and Evidence Certainty

**Figure 3:**
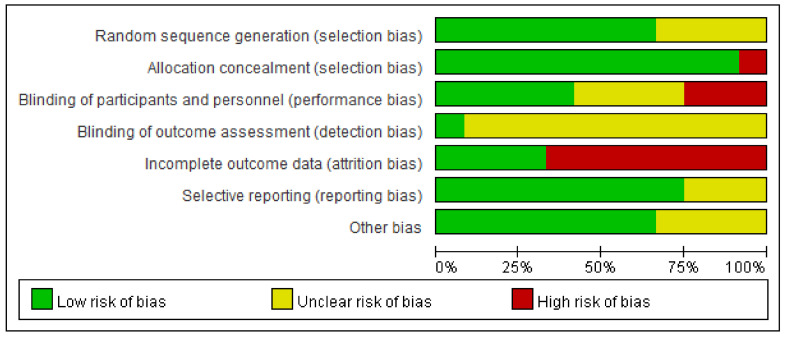
Summarized risk-of-bias graph for all included studies.

The studies’ risk of bias is shown in Figure 3. They were generally at low risk of bias for most domains including allocation concealment (92%), selective reporting (75%), and random sequence generation (67%) and at an unclear risk of bias for the blinding of the outcome assessment (92%). The blinding of the participants and investigators was at low risk in five studies (42%), unclear in four (33%), and at high risk in three (25%). The completeness of the outcome reporting was at low risk in four studies (33%) and high risk in eight (67%).

The evidence certainty for the primary outcomes including muscle mass, grip strength, one-repetition maximum in leg strength, walking speed, and the timed up and go test were rated from moderate to very low according to GRADE (Table 2).

### 3.3. Effects of n-3 PUFAs

A summary of the effects of the n-3 PUFAs on muscle mass, muscle strength, or muscle performance and the quality of the evidence according to GRADE are shown in Table 2.

**Figure 4:**
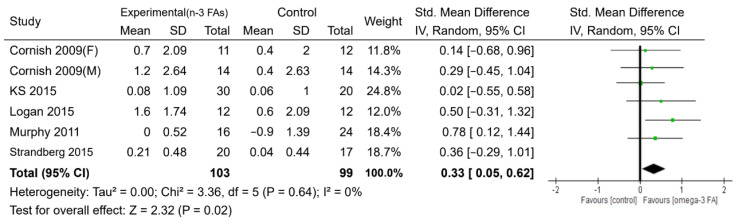
Forest plot of the effect of n-3 PUFA supplementation on muscle mass. IV: inverse-variance method. Random: random effect. Weight (in %), the influence of an individual study on the pooled result.

**Figure 5:**
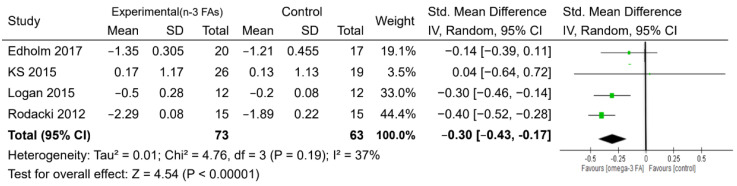
Forest plot of the effect of n-3 PUFA supplementation on the timed up and go test result. IV: inverse-variance method. Random, random effect. Weight (in %): the influence of an individual study on the pooled result.

For muscle mass, six studies with 202 participants reported measures of skeletal muscle mass based on the use of dual-energy X-ray absorptiometry, bioelectrical impedance analysis, or computed tomography, with intervention periods ranging from 10 to 24 weeks (Table 1 and Table 2). There was evidence to support a beneficial effect of n-3 PUFA supplementation on the increase in skeletal muscle mass, compared with the control, with a small-to-moderate effect (SMD = 0.33, *p* < 0.05) (Figure 4). Regarding muscle strength, the administration of n-3 PUFAs did not increase handgrip strength, the one-repetition maximum strength of the leg, and the walking speed compared with the controls (Supplementary Figure S1). However, the n-3 PUFAs group showed better performance (took less time) on the timed up and go test than the control group (MD = −0.30 s, *p* <0.05) (Figure 5).

**Figure 6:**
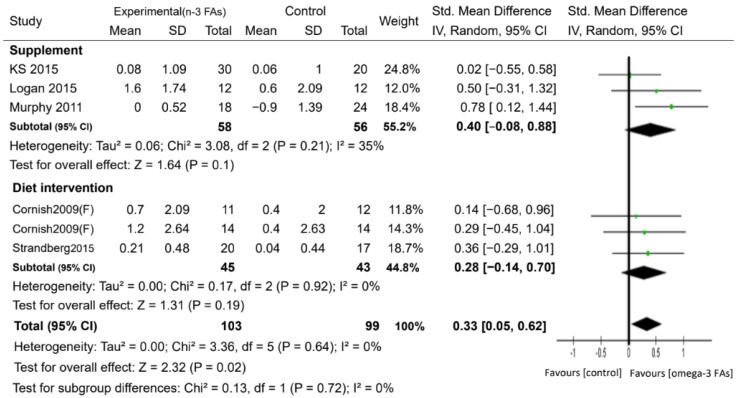
Forest plots of the included studies assessing the effect of n-3 PUFA supplementation on muscle mass categorized by the administration form of n-3 PUFAs. IV: inverse-variance method. Random: random effect. Weight (in %): the influence of an individual study on the pooled result.

Potential sources of heterogeneity between the studies were explored for the outcomes of muscle mass and walking speed. For muscle mass, we found no significant difference in the muscle mass between the subjects in the PUFA supplementation and dietary PUFA groups (Figure 6). We conducted a subgroup analysis to assess the effect of n-3 PUFA supplementation on muscle mass according to sex (Supplementary Figure S2a). There was no significant difference in the muscle mass between the n-3 PUFA group and the control group in both 61 females [25,35,38] and 14 males [25]. On the other hand, only one study by Cornish et al. [25] evaluated male participants according to n-3 PUFAs versus control. They also reported that n-3 PUFAs did not lead to an increase in muscle mass. When the subgroup analysis was based on the dosage of n-3 PUFAs, we found that only participants who received over 2 g/day of n-3 PUFAs had a significant increase in muscle mass compared to the control group (SMD = 0.67, *p* < 0.05) (Supplementary Figure S2b). By contrast, only one study [35] showed that participants who received n-3 PUFAs below 2 g/day exhibited no significant difference in muscle mass.

For walking speed, we found that studies with a follow-up duration of at least 24 weeks showed significant improvements in the walking speeds of subjects who received n-3 PUFAs, compared with the control group (SMD = 1.78, *p* < 0.05) while those with follow-up periods less than 24 weeks failed to show significant differences in walking speed (Supplementary Figure S3c). Four studies evaluated a female group, and one study by Cornish et al. [25] evaluated a male group; the results indicated that females or males who received n-3 PUFAs showed no improvement in walking speed (Supplementary Figure S3a). Finally, when the subjects were stratified in combination with a resistance exercise intervention, the results showed no difference in walking speed (Supplementary Figure S3b).

### 3.4. Sensitivity Analysis and Publication Bias

To appraise the stability of the results, sensitivity analyses were carried out using the leave-one-out approach and recalculating the summary SMD. The results show that our findings are robust for muscle mass, handgrip strength, the one-repetition maximum strength of the leg, walking speed, and timed up and go performance (Supplementary Figure S4b–e). However, for muscle mass performance, the results show that the beneficial effect of n-3 PUFAs on skeletal muscle mass was not observed when the study of Murphy et al. [37] on cancer patients was excluded from the meta-analysis (Supplementary Figure S4a). In addition, no included trial was at low risk of bias, thus precluding the performance of the preplanned sensitivity analysis. The shapes of the funnel plots were symmetric, indicating that the publication bias was low in the meta-analysis (*p*-value of Egger’s test > 0.05) (Supplementary Figure S5).

## 4. Discussion

Nutritional studies for sarcopenia-related performances had mostly focused on investigating the effects of protein supplementation. Even though studies had discussed n-3 PUFAs, outcomes with strong evidence or clear conclusions were seldom presented [15,16]. Our findings with 10 RCTs and 552 elderly participants showed that n-3 PUFA supplementation was associated with an increase in muscle mass by ~0.33 kg for the elderly, especially when more than 2 g/day of n-3 PUFAs was given. In terms of muscle strength, we found that n-3 PUFA supplementation did not elicit greater handgrip strength or one-repetition maximum strength of the leg. For muscle performance, n-3 PUFA administration slightly enhanced performance in the timed up and go test compared to that for the controls and facilitated a faster walking speed when administered for more than 24 weeks.

Our present finding that increased n-3 PUFA supplementation has a positive health effect on muscle mass is consistent with previous reports indicating that dietary ω-3 fatty acids increase the rate of muscle protein synthesis in older subjects [19], and suppresses the inflammation cascade in patients with Duchenne muscular dystrophy [40]. Muscle mass is maintained by a balance between muscle protein synthesis and breakdown. The supplementation of n-3 PUFAs increases the n-3 fatty acid composition of the phospholipids in the skeletal muscle membranes. Several muscle synthesis mechanisms involving n-3 PUFAs have been proposed, including the induction of the mTORC1-p70S6K1 signaling pathway, which leads to increased protein synthesis [41] and the downregulation of proteasome expression, thus suppressing muscle protein catabolism [42]. Concordantly, both animal [43] and human [19,40] studies have shown that n-3 PUFA supplementation enhances amino acid- and insulin-mediated increases in the rates of muscle protein syntheses. As for n-3 PUFA sources, there is a report of a strong association between EPA supplementation, increased plasma EPA levels, and elicited gains in the muscle mass of patients with non-small cell lung cancer [37]. This is consistent with evidence that lower EPA levels are associated with lower muscle mass, strength and function [44]. According to Logan et al. and Muphy et al.’s studies [36,37], when more than 2 g of n-3 PUFAs were supplied, an additional ~0.67 kg muscle mass was retained than when 1.1 g of n-3 PUFAs were given [35] according to our subgroup analysis. Although we cannot identify the effect for the elderly on taking only DHA or EPA or both based on limited studies, our meta-analysis still found that supplementation with both types of n-3 PUFAs (EPA and/or DHA) elicits a ~ 0.33 kg increase in muscle mass. Therefore, we suggest that n-3 PUFA supplementation has potential as an efficacious nutrition-based preventive or therapeutic strategy to combat the loss of muscle mass in the elderly, and the optimal dosing pattern for n-3 PUFAs for sarcopenia-associated performance needs to be confirmed by a large-scale RCT in the future.

The original definition of sarcopenia by the European Working Group on Sarcopenia in Older People (EWGSOP) was the “presence of both low muscle mass + low muscle function (strength or performance)” [3]. The recently updated consensus EWGSOP guideline for the case-finding and diagnosis of sarcopenia (EWGSOP2) focused on low muscle strength (and not low muscle mass) as a principal feature of sarcopenia [45]. Grip strength and chair stand measures are widely used for measuring muscle strength. Fish intake related to muscle strength in observational studies are not consistent [22,46]. Smith et al. [21] supplied 1.86 g of EPA and 1.5 g of DHA for older adults (aged 68.3 years old) for 6 months and observed positive outcomes in terms of sarcopenia-related performances. However, 1.3 g of n-3 PUFAs for 3 months failed to generate positive outcomes [35], and even 3-years treatment with low dose EPA (225 mg) and DHA (800 mg) resulted in no significant difference in chair stand performance according to resent RCT research [23]. We did not observe any improvement in muscle strength following the n-3 PUFA supplementation in this meta-analysis. The doses of the supplements, durations of the interventions, methodologies for assessing body composition, and characteristics of the study populations might have contributed to the inconsistency in the findings between studies.

Commonly used physical performance measures include timed up and go test performance and the gait speed. The timed up and go test is a functional mobility test that estimates the time it takes the participant to rise from an arm chair, walk 3 m away, return, and then sit down. We observed an improvement in the timed up and go test performance following n-3 PUFA supplementation, by 0.3 s, in our results. Because the percentage weight indicates the influence of an individual study on the pooled result, a larger study with a more precise effect size estimate gets a higher weight, and a smaller study gets a lower weight. For example, among these four trails (Figure 5), the percentage weight of Rodacki et al.’s trail [20], with a larger sample size, was 44.4%, which is higher than that (3.5%) of Krzyminska-Siemaszko et al.’s trail [35], with a smaller sample size. Recently, Sang-Rok’s RCT trail [47] found that fish oil consumption combined with resistance training improved the strength and physical function indicators such as Timed Up and Go performance in community-dwelling older adults. Consistent with our findings, the three-city study showed that higher plasma concentrations of long-chain n-3 PUFAs were associated with a lower proportion of individuals with slow gait speed [48], but only among the elders receiving at least 6-month of n-3 PUFA supplementation in subgroups from two studies [34,39]. We found that compared with the control group, n-3 PUFAs improved the walking speed by 1.78 m/sec. This change is clinically relevant in the context of aging [49].

The limitations of this meta-analysis include the limited numbers of relevant trials. Secondly, the results were heterogeneous. The differences in the n-3 PUFAs dosage and constitution, frequency of administration, follow-up duration, study cohort and control group in each trial could have led to different supplementation efficacy. Even though we used a random-effects model in the meta-analysis, there are still possible residual confounders such as the baseline nutritional status of the cohort in each trial, which was not described. Third, data regarding the physical exercise regimens were reported for only five RCTs and thus, it was not possible to isolate the effect of n-3 PUFAs intake alone. Finally, it was mentioned that the beneficial effect of n-3 PUFAs on muscle mass was not observed after excluding from the meta-analysis a study on patients with cancer [37]. This raises the question of a cohort-/sample-specific effect of n-3 PUFAs on muscle mass and the importance of further research in this respect to clarify the issue.

## 5. Conclusions

The present meta-analysis based on 10 studies found moderate evidence for the beneficial effect of n-3 PUFAs on muscle mass, especially for those taking supplements at more than 2 g/day. We also observed that a long period of n-3 PUFA supplementation may improve walking speed. The appropriate supplementation of n-3 PUFAs may have benefits on muscle mass and performances among the elderly.

## Figures and Tables

**Table 1:** Study characteristics of the included trials.

Author (Year)	Country	Age (Years, Mean)	Subjects (n)	Sex (% Female)	Duration of Intervention (Weeks)	Exercise	Intervention: g/day, *Sources*	Outcome Measures
Cornish and Chilibeck (2009) [25]	Canada	65.4	Healthy (51)	45	12	RS	ALA:14 *Flax oil*	MM: lean tissue mass; MS: one-repetition maximum leg strength
Murphy et al. (2011) [37]	Canada	63.3	NSCLC (40)	48	~10	NA	EPA:2.2 *FO capsules or liquid*	MM: whole-body skeletal muscle
Rodacki et al. (2012) [20]	Brazil	64.1	Healthy (45)	100	12-20	RS	EPA:0.4 DHA:0.3 *FO capsules*	MS: knee flexor and extensor peak torque; MP: chair rising, Sit and reach, foot up and go, 6-min walk
Hutchins-Wiese et al. (2013) [34]	USA	75	Postmenopausal women (126)	100	24	NA	EPA:0.72 DHA:0.48 *FO capsules*	MS: hand grip; MP: walking speed, 8 foot walk, repeated chair rises
Krzyminska-Siemaszko et al. (2015) [35]	Poznan	74.9	Healthy (53)	100	12	NA	EPA:0.66 DHA:0.44 *FO capsules*	MM: ALM index, skeletal muscle mass, fat-free mass; MS: hand grip; MP: timed up and go test, 4 m walking test
Logan et al. (2015) [36]	Canada	66.1	Healthy (24)	68	12	NA	EPA:2 DHA:1 *FO capsules*	MM: lean mass; MS: grip strength; MP: timed up and go test, 30 s sit to stand
Strandberg et al. (2015) [38]	Sweden	67.7	Healthy (63)	100	24	RS	n-6/n-3 <2	MM: leg lean mass; MS: one-repetition maximum leg strength
Grenon et al.(2015) [33]	USA	68.5	PAD (80)	2	4	NA	EPA:2.6 DHA:1.8 *FO capsules*	MP: walking distance, walking speed
Smith et al. (2015) [21]	USA	68.3	Healthy (60)	66	24	NA	EPA:1.86 DHA:1.5 *FO pill with EE form*	MM: thigh muscle volume; MS: handgrip strength, one-repetition maximum leg strength
Strike et al. (2016) [39]	UK	66.8	Postmenopausal women (29)	100	24	NA	EPA:0.16 DHA:1.0 *FO capsules*	MP: habitual walking speed
Da Boit et al.(2017) [31]	UK	70.6	Healthy (58)	46	18	RS	EPA:2.1 DHA:0.6 *FO capsules with* *TG form*	MM: muscle ACSA; MS: maximal isometric torque; MP: 4 m walk time, chair-rise time
Edholm et al. (2017) [32]	Sweden	67.7	Healthy (63)	100	24	RS	n-6/n-3 <2	MM: whole body lean mass; MS: knee extension peak power one-repetition maximum; MP: five sit-to-stand, single-leg-stance tests, timed up and go Test

Abbreviations: ACSA; anatomic cross-sectional area; ALA; a-linolenic acid; NSCLC; non-small-cell lung cancer; DHA; docosahexaenoic acid; EPA; eicosapentaenoic acid; EE; ethyl esters; FO; fish oil; MM; muscle mass; MP; muscle performance; MS; muscle strength; NA, not available; PAD; peripheral artery disease; RS; resistance training; TG; triglyceride.

**Table 2:** Summary effects of n-3 PUFAs on the outcomes of interest among the included studies and quality evidence of the Grading of Recommendations Assessment, Development and Evaluation (GRADE).

Outcome	No. of Studies	No. of Participants	Statistical Method	Effect Estimate	*p*-Value	Heterogeneity (I^2^)	Certainty of the Evidence (GRADE)
Muscle mass (kg)	6	202	SMD. Random	0.33 (0.05, 0.62)	0.02	0%	⊕⊕⊕◯ MODERATE
Grip strength (kg)	3	97	SMD. Random	0.53 (−0.64, 1.69)	0.37	85%	⊕◯◯◯ VERY LOW
One-repetition maximum leg strength (kg)	3	88	SMD. Random	−0.15 (−0.93, 0.62)	0.70	69%	⊕◯◯◯ VERY LOW
Walk speed (m/sec)	5	251	SMD. Random	0.81 (−0.05, 1.67)	0.06	88%	⊕◯◯◯ VERY LOW
Time up and go test (s)	4	136	MD. Random	−0.30 (−0.43, −0.17)	<0.0001	37%	⊕◯◯◯ VERY LOW

Abbreviations: SMD: standardized mean difference; MD: mean difference.

## Supplementary Materials

The following are available online at https://www.mdpi.com/2072-6643/12/12/3739/s1. Figure S1. Forest plots of the effect of n-3 PUFA supplementation on handgrip (a), one-repetition maximum strength of the leg (b), and walking speed (c). Figure S2. Forest plots of the included studies assessing the effect of n-3 PUFA supplementation on muscle mass categorized by sex (a) and the dosage of n-3 PUFAs (b). Figure S3. Forest plots of the included studies assessing the effect of n-3 PUFA supplementation on walking speed categorized by sex (a), resistance training intervention (b), and the duration of supplementation (c). Figure S4. Sensitivity analyses by muscle mass (a), handgrip strength (b), one-repetition maximum strength of the leg (c), walking speed (d), and the timed up and go test result (e). Figure S5. Charts of Egger’s test of muscle mass (a), handgrip strength (b), one-repetition maximum strength of the leg (c), walking speed (d), and the timed up and go test result (e).
