# Supplementary material for: Effects of Omega-3 Fatty Acids on Muscle Mass, Muscle Strength and Muscle Performance among the Elderly: A Meta-Analysis
Source: Nutrients. 2020 Dec 4;12(12):3739. doi: 10.3390/nu12123739 (PMC7761957; doi:10.3390/nu12123739)
Supplement: Supplementary file 1 [file nutrients-12-03739-s001.pdf]

(a)

Study or Subgroup	omega-3 FA Mean	SD	Total	Control Mean	SD	Total	Weight	Std. Mean Difference IV, Random, 95% CI
KS2015	-0.17	1.91	30	0.85	3.14	19	35.8%	-0.41 [-0.99, 0.17]
Logan 2015	1.6	4.32	12	-0.3	3.26	12	33.0%	0.48 [-0.33, 1.29]
Strike2016	1.6	1.25	12	-0.3	0.94	12	31.2%	1.66 [0.71, 2.61]
**Total (95% CI)**			**54**			**43**	**100.0%**	**0.53 [-0.64, 1.69]**

Heterogeneity: Tau² = 0.90; Chi² = 13.68, df = 2 (P = 0.001); I² = 85%
Test for overall effect: Z = 0.89 (P = 0.37)

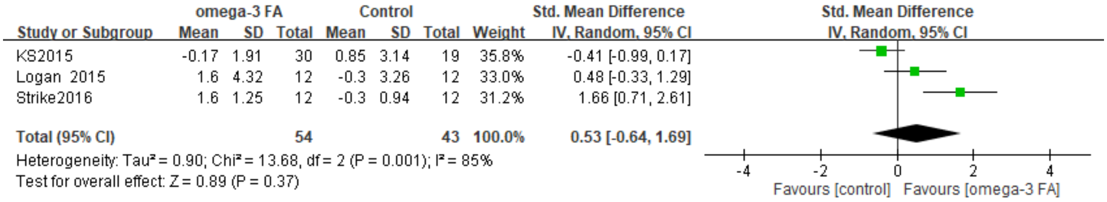

(b)

Study or Subgroup	Experimental Mean	SD	Total	Control Mean	SD	Total	Weight	Std. Mean Difference IV, Random, 95% CI
Cornish 2009 (F)	23.58	5.17	11	29.09	4.835	12	29.9%	-1.06 [-1.95, -0.18]
Cornish 2009 (M)	25.4	3.875	14	24.55	3.79	14	33.7%	0.22 [-0.53, 0.96]
Strandberg 2015	10.5	2.06	20	9.8	3.28	17	36.4%	0.25 [-0.39, 0.90]
**Total (95% CI)**			**45**			**43**	**100.0%**	**-0.15 [-0.93, 0.62]**

Heterogeneity: Tau² = 0.32; Chi² = 6.36, df = 2 (P = 0.04); I² = 69%
Test for overall effect: Z = 0.38 (P = 0.70)

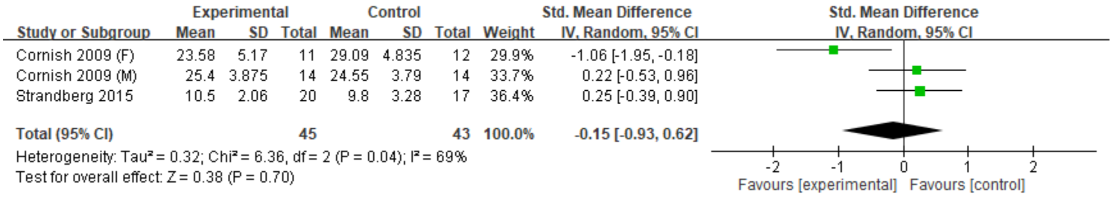

(c)

Study or Subgroup	omega-3 FA Mean	SD	Total	Control Mean	SD	Total	Weight	Std. Mean Difference IV, Random, 95% CI
Da Boit 2017 (F)	0.16	0.17	13	0.12	0.17	10	19.1%	0.23 [-0.60, 1.05]
Da Boit 2017 (M)	0.27	0.19	14	0.22	0.16	13	19.6%	0.28 [-0.48, 1.03]
Hitchins-Wiese 2013	0.03	0.03	85	-0.03	0.04	41	21.9%	1.78 [1.34, 2.21]
KS2015	-0.19	0.55	29	-0.19	0.725	19	21.0%	0.00 [-0.58, 0.58]
Strike2016	0.06	0.07	15	-0.06	0.06	12	18.4%	1.77 [0.85, 2.68]
**Total (95% CI)**			**156**			**95**	**100.0%**	**0.81 [-0.05, 1.67]**

Heterogeneity: Tau² = 0.83; Chi² = 33.32, df = 4 (P < 0.00001); I² = 88%
Test for overall effect: Z = 1.85 (P = 0.06)

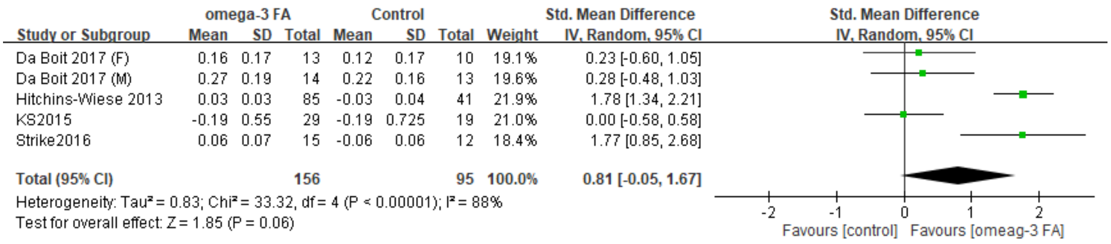

Supplementary Figure S1. Forest plots of the effect of n-3 PUFA supplementation on handgrip (a), one-repetition maximum strength of the leg (b), and walking speed (c).

(a)

Study or Subgroup	Experimental Mean	Experimental SD	Experimental Total	Control Mean	Control SD	Control Total	Weight	Std. Mean Difference IV, Random, 95% CI
**2.2.1 Female**								
Cornish 2009 (F)	0.7	2.09	11	0.4	2	12	11.8%	0.14 [-0.68, 0.96]
KS2015	0.08	1.09	30	0.06	1	20	24.8%	0.02 [-0.55, 0.58]
Strandberg 2015	0.21	0.48	20	0.04	0.44	17	18.7%	0.36 [-0.29, 1.01]
**Subtotal (95% CI)**			**61**			**49**	**55.4%**	**0.16 [-0.22, 0.54]**
Heterogeneity: Tau² = 0.00; Chi² = 0.60, df = 2 (P = 0.74); I² = 0%								
Test for overall effect: Z = 0.83 (P = 0.41)								
**2.2.2 male**								
Cornish 2009 (M)	1.2	2.64	14	0.4	2.63	14	14.3%	0.29 [-0.45, 1.04]
**Subtotal (95% CI)**			**14**			**14**	**14.3%**	**0.29 [-0.45, 1.04]**
Heterogeneity: Not applicable								
Test for overall effect: Z = 0.77 (P = 0.44)								
**2.2.3 Both**								
Logan 2015	1.6	1.74	12	0.6	2.09	12	12.0%	0.50 [-0.31, 1.32]
Murphy 2011	0	0.52	16	-0.9	1.39	24	18.4%	0.78 [0.12, 1.44]
**Subtotal (95% CI)**			**28**			**36**	**30.3%**	**0.67 [0.16, 1.18]**
Heterogeneity: Tau² = 0.00; Chi² = 0.27, df = 1 (P = 0.60); I² = 0%								
Test for overall effect: Z = 2.57 (P = 0.01)								
**Total (95% CI)**			**103**			**99**	**100.0%**	**0.33 [0.05, 0.62]**
Heterogeneity: Tau² = 0.00; Chi² = 3.36, df = 5 (P = 0.64); I² = 0%								
Test for overall effect: Z = 2.32 (P = 0.02)								
Test for subgroup differences: Chi² = 2.48, df = 2 (P = 0.29), I² = 19.5%								

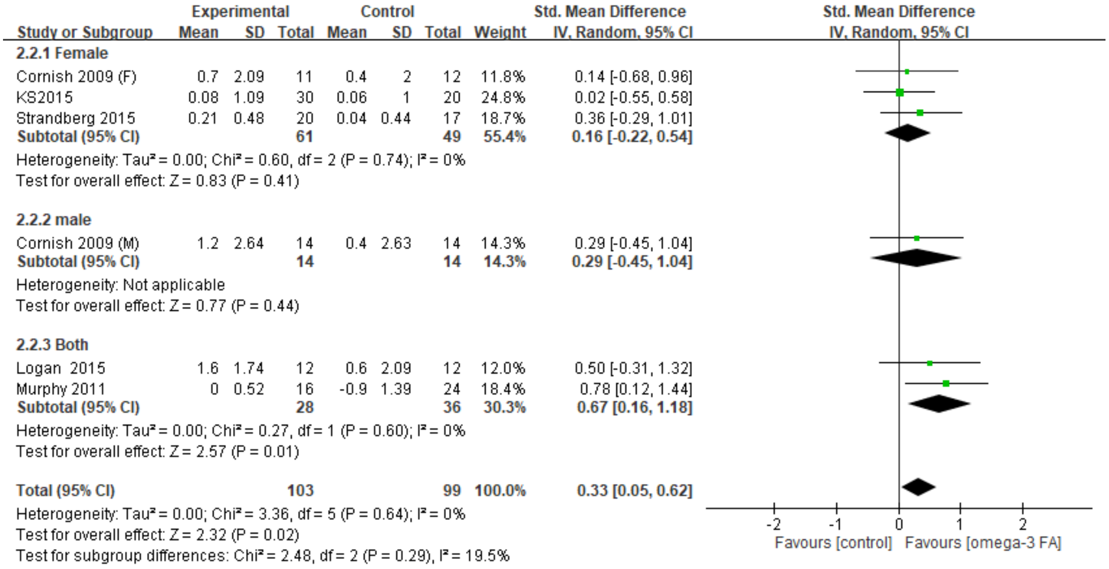

(b)

Study or Subgroup	experimental(n-3 PUFAs) Mean	experimental(n-3 PUFAs) SD	experimental(n-3 PUFAs) Total	control Mean	control SD	control Total	Weight	Std. Mean Difference IV, Fixed, 95% CI
**2.2.1 over 2g**								
Logan 2015	1.6	1.74	12	0.6	2.09	12	21.7%	0.50 [-0.31, 1.32]
Murphy 2011	0	0.52	16	-0.9	1.39	24	33.3%	0.78 [0.12, 1.44]
**Subtotal (95% CI)**			**28**			**36**	**55.0%**	**0.67 [0.16, 1.18]**
Heterogeneity: Chi² = 0.27, df = 1 (P = 0.60); I² = 0%								
Test for overall effect: Z = 2.57 (P = 0.01)								
**2.2.2 below 2g**								
KS2015	0.08	1.09	30	0.06	1	20	45.0%	0.02 [-0.55, 0.58]
**Subtotal (95% CI)**			**30**			**20**	**45.0%**	**0.02 [-0.55, 0.58]**
Heterogeneity: Not applicable								
Test for overall effect: Z = 0.06 (P = 0.95)								
**Total (95% CI)**			**58**			**56**	**100.0%**	**0.38 [-0.00, 0.76]**
Heterogeneity: Chi² = 3.08, df = 2 (P = 0.21); I² = 35%								
Test for overall effect: Z = 1.95 (P = 0.05)								
Test for subgroup differences: Chi² = 2.81, df = 1 (P = 0.09), I² = 64.4%								

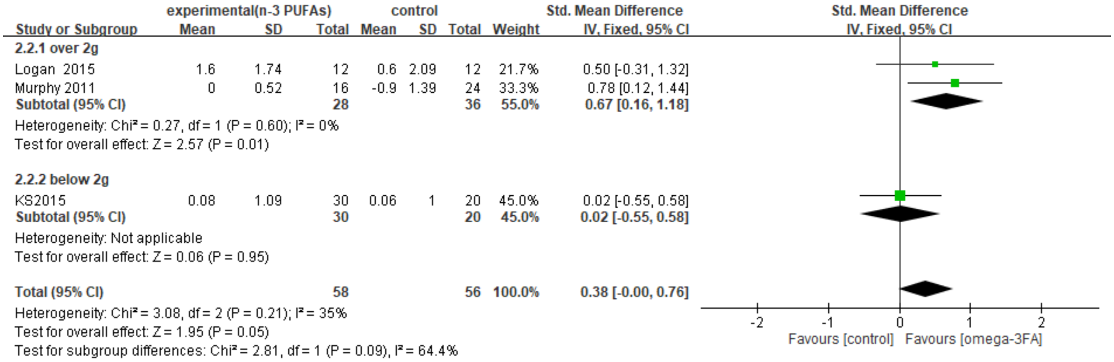

Supplementary Figure S2. Forest plots of the included studies assessing the effect of n-3 PUFA supplementation on muscle mass categorized by sex (a) and the dosage of n-3 PUFAs (b).

(a)

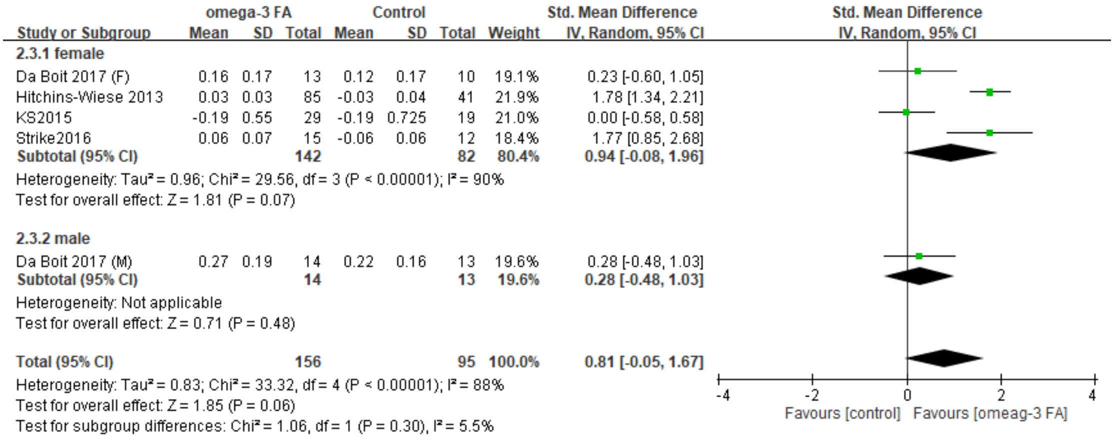

(b)

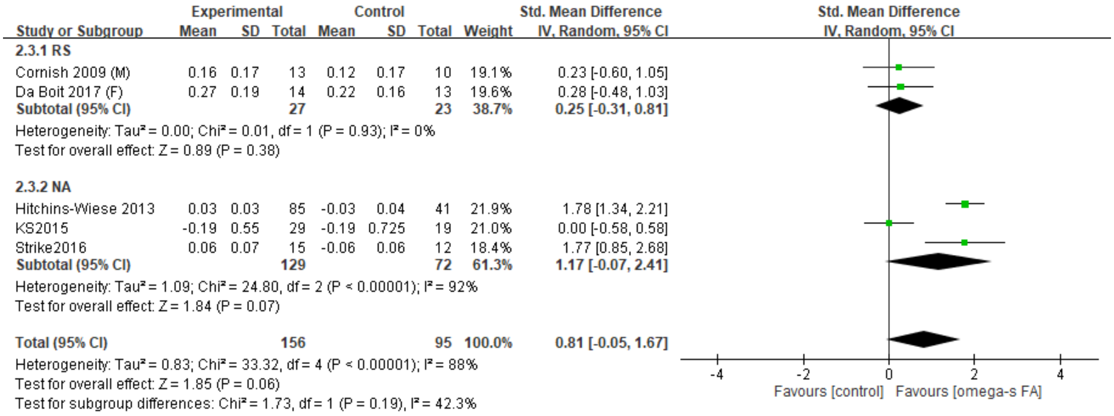

(c)

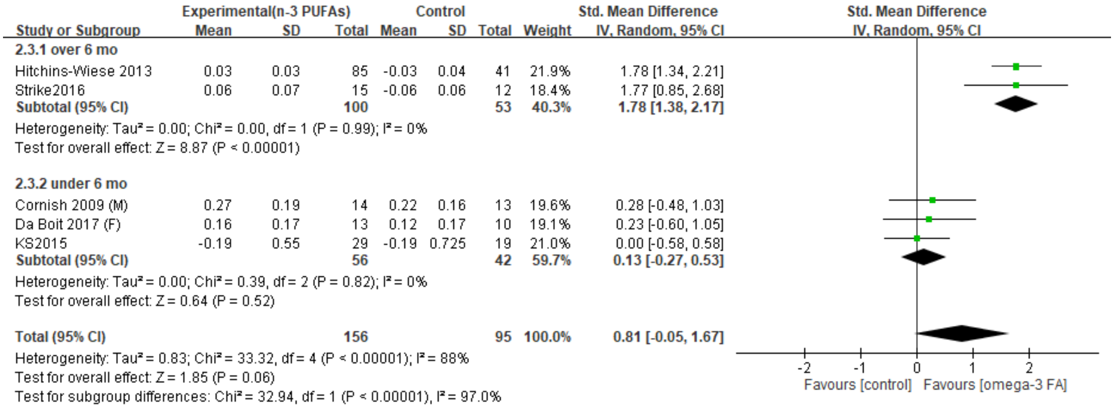

Supplementary Figure S3. Forest plots of the included studies assessing the effect of n-3 PUFA supplementation on walking speed categorized by sex (a), resistance training intervention (b), and the duration of supplementation (c).

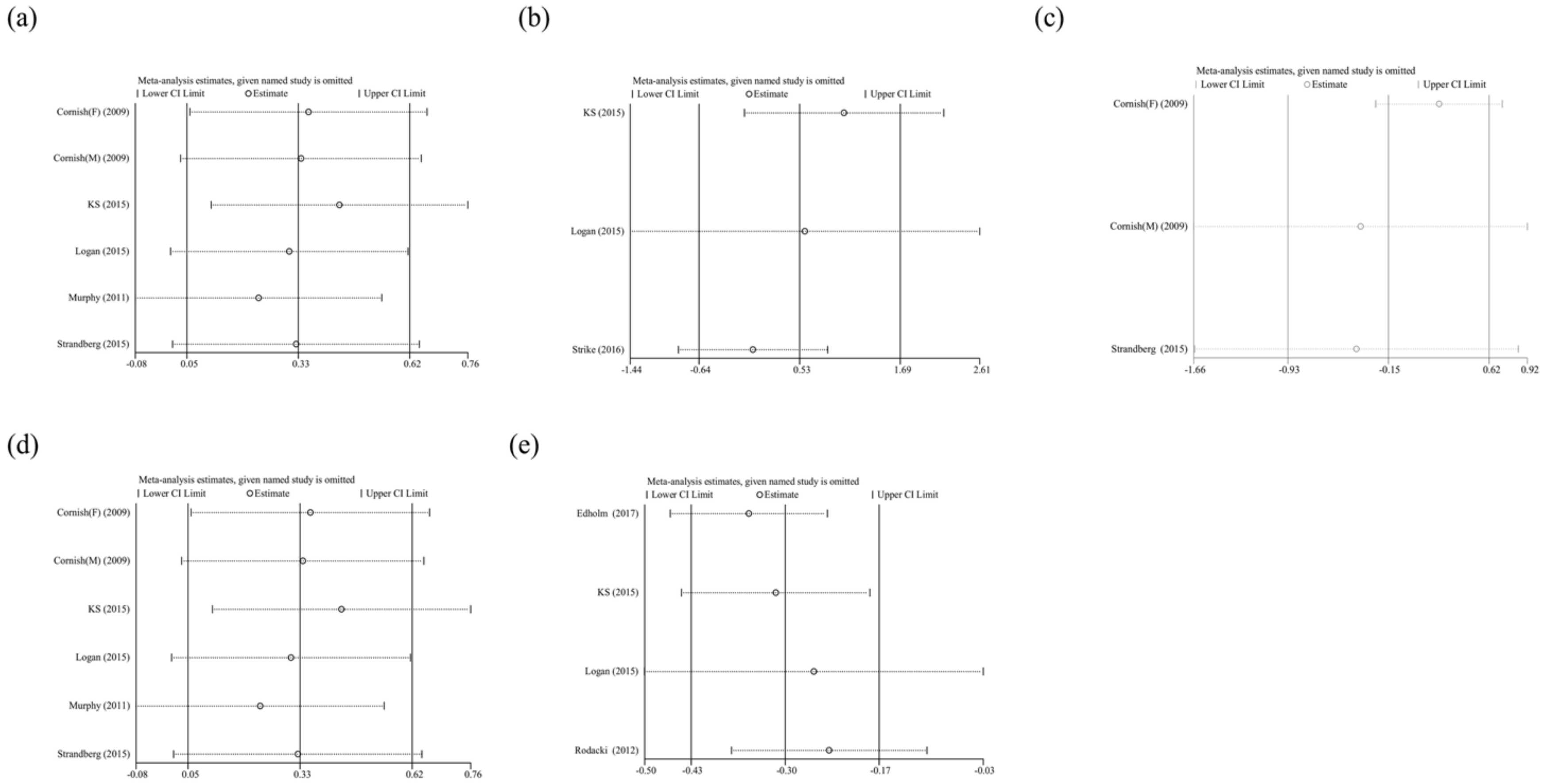

Supplementary Figure S4. Sensitivity analyses by muscle mass (a), handgrip strength (b), one-repetition maximum strength of the leg (c), walking speed (d), and the timed up and go test result (e).

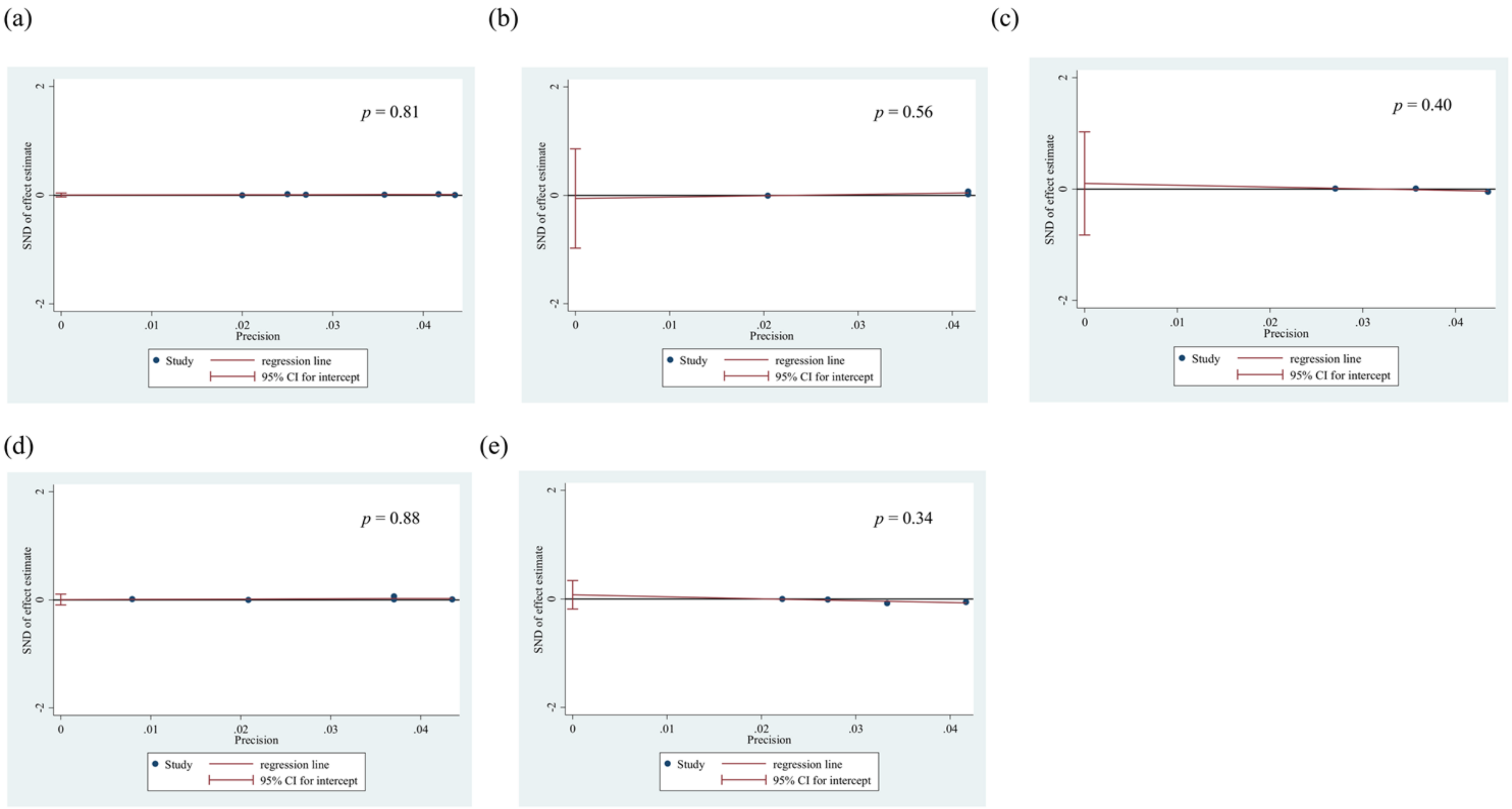

Supplementary Figure S5. Charts of Egger's test of muscle mass (a), handgrip strength (b), one-repetition maximum strength of the leg (c), walking speed (d), and the timed up and go test result (e).
